# Endocytosis‐Inspired Zwitterionic Gel Tape for High‐Efficient and Sustainable Underoil Adhesion

**DOI:** 10.1002/advs.202407501

**Published:** 2024-09-09

**Authors:** Yueman Tang, Mengjie Si, Yan‐jie Wang, Jiahui Zhou, Yuming Deng, Kaishun Xia, Zhen Jiang, Dong Zhang, Si Yu Zheng, Jintao Yang

**Affiliations:** ^1^ College of Materials Science & Engineering Zhejiang University of Technology Hangzhou 310014 P. R. China; ^2^ School of Materials Science and Engineering Tiangong University Tianjin 300387 P. R. China; ^3^ Department of Orthopedics, The Second Affiliated Hospital School of Medicine Zhejiang University Hangzhou 310009 P. R. China; ^4^ School of Mechanical Materials Mechatronic and Biomedical Engineering University of Wollongong Wollongong NSW 2522 Australia; ^5^ Department of Biomedical Engineering Georgia Institute of Technology Atlanta GA 30332 USA

**Keywords:** sustainable materials, tough gel, underoil adhesion, wet adhesive, zwitterion

## Abstract

Marine oil exploration is important yet greatly increases the risk of oil leakage, which will result in severe environment pollution and economic losses. It is an urgent need to develop effective underoil adhesives. However, realizing underoil adhesion is even harder than those underwater, due to the stubborn attachment of a highly viscous oil layer on target surface. Here, inspired by endocytosis, a tough gel tape composed of zwitterionic polymer network and zwitterionic surfactants is developed. The amphiphilic surfactants can form micelle to capture the oil droplets and transport them from the interface to gel via electrostatic attraction of polymer backbone, mimicking the endocytosis and achieving robust underoil adhesion. Benefiting from the oil‐resistance of polymer backbone, the gel further realizes a combination of i) long‐term adhesion with high durability, ii) repeated adhesion in oil, and iii) renewable adhesion efficiency after exhausted use. The tape exhibits an ultra‐high adhesive toughness of 2446.86 J m^−2^ to stainless steel in silicone oil after 30 days' oil‐exposure; such value of adhesive toughness surpasses many of those achieved in underwater adhesion and is greater than underoil adhesion performance of commercial tape. The strategy illustrated here will motivate the design of sustainable and efficient adhesives for wet environments.

## Graphical abstract

Inspired by endocytosis, a gel adhesive based on zwitterionic polymer and amphiphilic surfactant with similar structure to phospholipid is designed. The obtained gel tape shows 1) durable adhesion in each single use, 2) repeated use potential, and 3) function regeneration after the exhausted use. The strategy illustrated here will motivate the design of sustainable and efficient adhesives for wet environments.

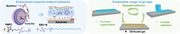

## 1 Introduction

As one of the most important energy resources, oil is the blood of the global economy and manufacturing industry. Due to the intensification of global energy crisis, the oil exploration gradually shifts from land to ocean, leading to the increasing risks of oil leakage, which not only threatens the marine ecosystem, but causes enormous economic loss.^[^ ^1^, ^2^, ^3^ ^]^ Therefore, it is highly urgent to develop effective underoil adhesives. However, achieving robust adhesion in wet environment is always a hard task, because the liquid layer adhered on the target surface will hinder the molecular bridging between the adhesive and adherend.^[^ ^4^, ^5^, ^6^, ^7^, ^8^, ^9^, ^10^ ^]^ Although great progress has been made in underwater adhesion,^[^ ^11^, ^12^, ^13^, ^14^, ^15^, ^16^, ^17^, ^18^, ^19^ ^]^ realizing robust underoil adhesion is much beyond that. Compared to water: i) the oil usually possesses much lower surface tension and much higher viscosity,^[^ ^20^, ^21^, ^22^ ^]^ making it much more difficult to be removed from the interface; ii) the sticky oil can easily absorb on the adhesive and even swell it, making the adhesive too slippery to realize robust adhesion. Therefore, achieving robust underoil adhesion is important yet still in its preliminary stage.^[^ ^23^, ^24^, ^25^, ^26^ ^]^

Up to now, some interesting methods have been proposed to realize oil removal and underoil adhesion. For example, Liu et al. developed a pressure sensitive adhesive with rough and drainage surface; the oil existed in the interface would be squeezed out via the drainage channel under pressure to realize underoil adhesion.^[^ ^27^ ^]^ Wang et al. developed an underoil adhesive containing water/oil dual‐soluble “mediator” solvent, which triggers turbulent liquid replacement to expel oil out of the interface.^[^ ^25^ ^]^ These works provide great strategies for underoil adhesion, yet mainly focus on the removal of oil layer. In practical, the sustainable usage of tape, including i) durable adhesion in each single use, ii) repeated use, and iii) function regeneration after the exhausted use are also highly required for elongating their service life and avoiding resource waste.^[^ ^28^, ^29^, ^30^, ^31^, ^32^, ^33^ ^]^ However, achieving durable and recycling adhesion in oil is quite difficult, because: i) the prevalent oil contamination may weaken the bulk strength of them, make their surface slippery, and lead to the failure of adhesion through gradually erosion; ii) the tape contaminated by oil will become too slippy to realize a second use, and the absorbed oil is difficult to remove from the tape, obstructing the repeated use and recycle of waste tape.^[^ ^24^ ^]^

The natural creatures have provided a lot of inspirations in designing high‐efficient adhesives.^[^ ^30^, ^34^, ^35^, ^36^, ^37^, ^38^, ^39^, ^40^, ^41^, ^42^, ^43^ ^]^ The creatures also show many clever designs in transportation of oily substances. For example, the cell is ingeniously designed for mass transfer, where the cell membrane plays a key role.^[^ ^44^ ^]^ The main component of the cell membrane, i.e., the phospholipid bilayer is assembled from amphiphilic phospholipid, which is composed of hydrophilic phosphocholine and hydrophobic aliphatic chain. The phospholipid bilayer is flowable and could deform to capture the extracellular oily nutrients by forming nutrients‐encapsulated vesicles (liposome) in situ, which are then transported into the cell via electrostatic interaction.^[^ ^45^, ^46^, ^47^, ^48^ ^]^ This process is known as endocytosis and provides a potential solution to transport the oil away from the interface.

**Figure 1:**
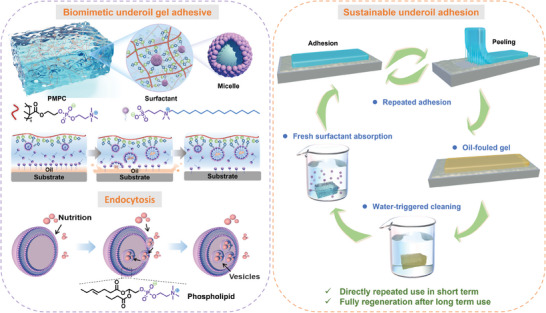
Schematic illustrating the design of endocytosis‐inspired underoil adhesives. Similar to the endocytosis of forming liposome in situ at cell membrane and uptake oily nutrients filled liposome into cell, the SB3‐16 arranged at oil‐water interface can capture the oil droplets by forming micelles, and the oil‐filled micelles are then transported into the gel though electrostatic interactions between SB3‐16 and PMPC. Besides, benefiting from the ultra‐hydrophilic nature of PMPC backbone, the gel tape achieves i) durable adhesion in each single use, ii) repeated use, and iii) functional regeneration after the exhausted use.

Inspired by endocytosis, we developed a tough gel tape with a combination of i) robust underoil adhesion with high durability, ii) repeated adhesion ability in oil, and iii) renewable adhesion efficiency for recycling use (**Figure** **1**). The gel is composed of poly(2‐methacryloyloxyethyl phosphorylcholine) (PMPC) network that shares similar groups to the phospholipid, and 3‐(N,N‐Dimethylpalmitylammonio) propanesulfonate (SB3‐16) zwitterionic surfactants with amphipathy like phospholipid. The obtained gel can efficiently remove the oil on the surface through the synergistic effect of PMPC and SB3‐16, with a similar mechanism to the endocytosis: i) the SB3‐16 arranged at gel surface will surround and capture the oil droplets by forming micelles in situ; ii) the oil‐encapsulated micelles are then transported into the gel via the electrostatic interactions between PMPC and SB3‐16. Meanwhile, the polar zwitterionic groups on PMPC can form diverse interactions to adherend and resist the oil attack, generating a high adhesive toughness of 2446.86 J m^−2^ to steel in highly viscous silicone oil after 30 days. Such value even outperforms most of the reported underwater adhesives and surpasses the underoil adhesion performance of commercial tapes. Furthermore, the adhesive toughness is almost maintained for 20 bonding‐peeling repeated cycles. Although the adhesion will decline after extremely repeated cycles, the adhesive toughness can be almost regenerated by water washing and re‐adsorption of SB3‐16, demonstrating the highly sustainable usage. We believe the strategy illustrated here will motivate the design of high‐efficient wet adhesives and sustainable adhesives.

## 2 Results and Discussion

### 2.1 Design and Characterization of Adhesives

The zwitterionic gel tape is prepared by one‐step polymerizing the glycerol/water solution of MPC in presence of SB3‐16 (Table S1, Supporting Information). The obtained gels are denoted as PMPC‐x%, where the x is the mass fraction of surfactant relative to MPC. The obtained gel shows good biocompatibility due to the biocompatible nature of zwitterionic polymer (Figure S1, Supporting Information).

**Figure 2:**
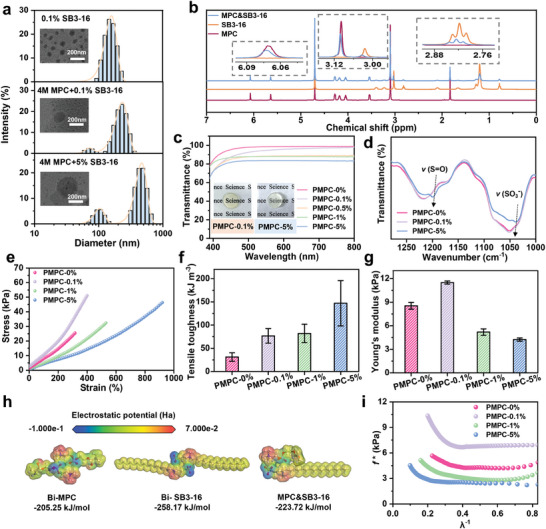
Characterization of the precursor and gel tape. a) Particle size distribution of precursor solutions with different compositions measured by DLS; the inset presents the corresponding TEM images. b) ^1^H NMR spectra of MPC, SB3‐16, and their mixture. c) Transmittance and d) FTIR spectra of the gels with different surfactant contents; the inset shows the appearance of the corresponding gel. e) Stress–strain curves of the gel with different surfactant contents; corresponding f) tensile toughness and g) Young's modulus. h) Binding energy of different molecular pairs from DFT calculations. i) The curves of reduced stress versus 𝜆^−1^. The results are represented as the mean ± SD (n = 3).

Considering the essential role of SB3‐16 surfactant, its aggregation state is first investigated. It is already known that the surfactants will arrange at surface of the phase preferentially to form a single layer due to its amphipathy; then, as the concentration of surfactants in bulk phase exceeds the critical micelle concentration (CMC), the saturated surfactants will assemble into micelle.^[^ ^49^, ^50^, ^51^, ^52^ ^]^ The CMC value of SB3‐16 in water at 25 °C is 0.000028 mol·dm^−3^,^[^ ^53^ ^]^ while addition of glycerol will slightly increase the CMC.^[^ ^54^ ^]^ To study the aggregation state of SB3‐16, the precursor solutions of i) 0.1% SB3‐16 without MPC, ii) 0.1% SB3‐16 with MPC and iii) 5% SB3‐16 with MPC were prepared for investigation. As presented in **Figure** **2**a, the results of transmission electron microscopy (TEM) and dynamic light scattering (DLS) both demonstrate the formation of micelles. The size of the micelles increases from 100 to 270 nm as the surfactant content increases from 0.1% to 5%. Besides, the micelle size of the groups with MPC is slightly larger than those without MPC, along with an additional size distribution in DLS result, indicating the MPC may participate the formation of micelles via forming electrostatic interactions to the SB3‐16. The molecular interaction between MPC and SB3‐16 is verified by ^1^H nuclear magnetic resonance (^1^H NMR), where the signals correspond to the H atoms adjacent to the anionic and cationic groups show obvious shift compared to those ones in individual components (Figure 2b; Figure S2, Supporting Information).

The UV–vis spectrum of the gels with different compositions is presented in Figure 2c and Figure S3 (Supporting Information). The PMPC‐0% and PMPC‐0.1% gels display high transparency with transmittance >90%; however, the gels containing more surfactants become a bit turbid, due to the existence of large‐sized micelles. The chemical structure of the gels is characterized by Fourier transform infrared spectroscopy (FTIR) (Figure 2d). As the surfactant content increases from 0% to 5%, the antisymmetric and symmetric stretching vibration peak of S═O bonds become more obvious (at 1199 and 1037 cm^−1^); besides, the position of S═O peaks appear at a slightly lower wavenumber compared to those of the typical S═O,^[^ ^55^ ^]^ indicating the formation of electrostatic interaction between SB3‐16 and PMPC again. The storage modulus *G'* of the gels with different compositions is higher than their loss modulus *G″* at the frequency of 0.1–100 rad s^−1^, demonstrating the solid‐like nature (Figure S4, Supporting Information).

The stress‐strain curves of the gels with different compositions are presented in Figure 2e. The tensile toughness of the gel will increase after introducing SB3‐16, yet the gel will significantly soften as the SB3‐16 is excessively added (Figure 2f,g). The enhancement in toughness should be ascribed to the hierarchical distribution of molecular interactions for efficient energy dissipation. The result of density functional theory (DFT) simulation shows that multiple interactions exist between the key components, where the value of binding energy fallows the order of Bi‐SB3‐16 > MPC‐SB3‐16>Bi‐MPC (Figure 2h). Therefore, the SB3‐16 micelles are connected to the PMPC network via electrostatic interactions and serve as physical cross‐links. During the loading process, the relative weak interactions in the system rupture first to dissipate energy, whereas the stronger ones remain to impart the elasticity. Excessive addition of surfactant will lower the pH of system and lead to the deionization of sulfonate/phosphate groups, weakening the electrostatic interactions and softening the gel (Figure S5, Supporting Information).

Furthermore, the mechanical characteristics of the gel can be better visualized by using the reduced stress model f^*^:^[^ ^56^, ^57^ ^]^

(1) $$f^{*}=\frac{\sigma }{\lambda -\lambda^{-2}}\;$$

where the 𝜎 and 𝜆 refer to the tensile stress and the elongation, respectively. It can be found that the reduced stress (related to the modulus) of PMPC‐0.1% gel is always higher than that of PMPC‐5% and PMPC‐0% gels across the full strain scale; meanwhile, the PMPC‐0.1% gel begin strain stiffen prior to the PMPC‐5% gel at a smaller strain, which is helpful to resist deformation against applied force similar to skin (Figure 2i).

### 2.2 Adhesion Performance of the Gel Tape

**Figure 3:**
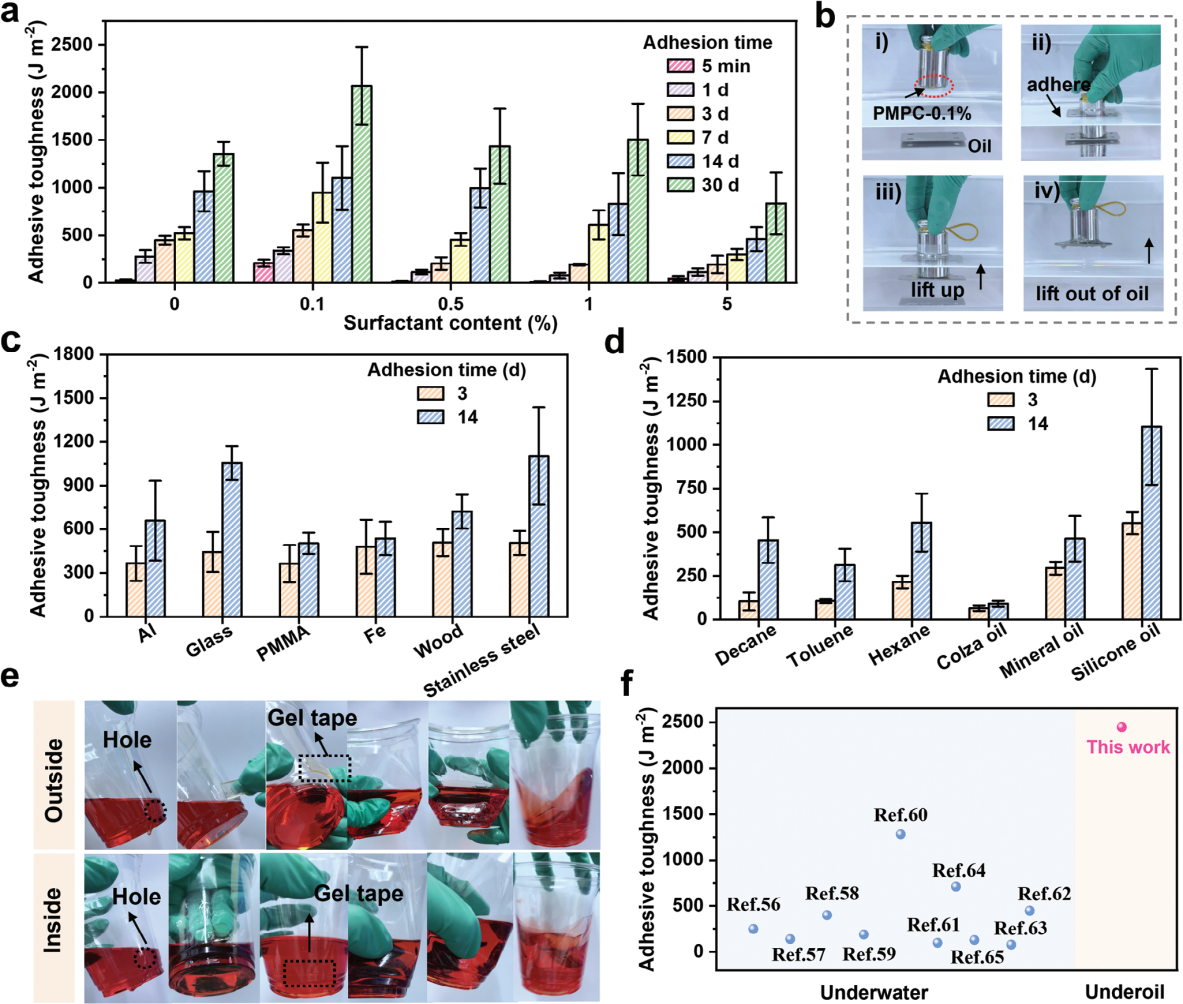
Adhesion performance of the gel tapes. a) Adhesive toughness of gels with different surfactant contents to the stainless steel surface after depositing in silicone oil for different intervals. b) The images to show the fast and robust underoil adhesion of PMPC‐0.1% gel by picking up a stainless steel underoil. Adhesive toughness of the PMPC‐0.1% gel to c) different materials in silicone oil and d) to stainless steel in different oily environments. e) Photographs to show the fast underoil sealing from outside and from inside with the assistance of PMPC‐0.1% gel. f) Comparison of the adhesive developed here with those reported in underwater adhesion. The results are represented as the mean ± SD (n = 3).

The adhesive performance of the gel is evaluated through the 90° peeling method^[^ ^58^ ^]^ (Figure S6, Supporting Information). To study the effect of surfactant amount on adhesion, the gels with different surfactant contents were prepared and attached to the oil‐wetted stainless steels for different adhesion time. Highly viscous silicone oil with viscosity of 1 000 mPa·S at 25 °C is selected as the model of oil. As shown in **Figure** **3**a, the adhesive toughness of all groups increases with elongated time, and exhibits excellent adhesion even after 1 month, suggesting outstanding durability. Among them, the PMPC‐0.1% gel exhibits the best adhesion performance, whose adhesive toughness achieves a high value of 249.26 J m^−2^ within short time (5 min) and increases constantly to 2446.86 J m^−2^ as the adhesion time increases to 30 days. Lower or higher surfactant content than 0.1% will sacrifice the adhesion performance partially. The underlying mechanism will be discussed in detail in the next section. Meanwhile, surfactant with varying alkyl chain length will also influence the adhesion toughness of gel adhesives. The adhesives prepared by the surfactants with longer alkyl chain length exhibit higher adhesion toughness. This is because longer alkyl chain could enlarge the hydrophilicity difference of the two sides of surfactant, promoting the formation of micelles (Figure S7, Supporting Information).^[^ ^50^ ^]^ Furthermore, the backbone of gel is replaced by other common polyzwitterions like poly(sulfobetaine methacrylate) (PSBMA) and poly(carboxybetaine methacrylate) (PCBMA). Both PSBMA gel tape and PCBMA gel tape containing 0.1% surfactants show higher underoil adhesion toughness than those of the pristine ones, demonstrating the universality of strategy (Figure S8, Supporting Information). Considering the PMPC with 0.1 wt.% SB3‐16 has superior underoil adhesion performance, PMPC‐0.1% gel is adopted for the subsequent tests.

As shown in Figure 3b and Figure S9 (Supporting Information), a metal weight can pick up stainless steel out of the silicone oil with the assistance of PMPC‐0.1% gel, demonstrating the rapid underoil adhesion. Two stainless steel plates adhered with PMPC‐0.1% gel can sustain a weight of 200 g beneath the oil, demonstrating robust underoil adhesion (Figure S10, Supporting Information). Besides the oil environment, the PMPC‐0.1% gel also shows good adhesion in air, which could maintain well for long time deposition due to the good water retention capacity imparted by glycerol (Figures S11 and S12, Supporting Information).

Furthermore, the PMPC‐0.1% gel exhibits excellent adhesion capacity to a wide range of materials in silicone oil at 14th day (Figure 3c), including the aluminum (Al, 852.35 J m^−2^), glass (1182.88 J m^−2^), poly(methyl methacrylate) (PMMA, 575.92 J m^−2^), iron (Fe, 607.56 J m^−2^), and wood (882.01 J m^−2^). Such wide‐ranging adhesion to diverse materials also works well in air (Figure S13, Supporting Information). Besides the type of materials, the PMPC‐0.1% gel also shows high adhesive toughness in diverse liquid oil environment at 14th day (Figure 3d), including decane (648.72 J m^−2^), toluene (419.46 J m^−2^), hexane (744.46 J m^−2^), colza oil (105.63 J m^−2^), mineral oil (564.2 J m^−2^). Benefiting from such high‐efficient adhesion, underoil sealing of broken container is realized. As shown in Figure 3e, a small hole is produced at the wall of a 420 mL plastic bottle, which is filled with red‐dyed silicone oil; the oil leakage is stopped immediately after applying PMPC‐0.1% tape to the leak point of bottle from inside or from outside. Besides, the tape keeps firm adhesion to bottle without peeling and oil leakage even as the bottle is significantly deformed or violently shaken once after adhesion, demonstrating the robust sealing capability. Then the bottle is immersed into a tank filled with undyed oil; as shown in Figure S14 (Supporting Information), the oil outside the bottle does not turns into red, indicating the successful sealing. To quantitatively evaluate the leakage, the oil outside the bottle is extracted to perform UV spectrum test; the resulting UV spectrum of the oil is comparable to that of pristine silicone oil, demonstrating the negligible leakage (Figure S15, Supporting Information). In addition, the sealing efficiency is well maintained without obvious oil leakage as the surrounding liquid outside the bottle is replaced with water (Figure S16, Supporting Information).

It is worth noting that although removing oil is more difficult than removing water due to the higher viscosity of oil, the underoil adhesive toughness achieved here outperforms many of those achieved underwater (Figure 3f).^[^ ^59^, ^60^, ^61^, ^62^, ^63^, ^64^, ^65^, ^66^, ^67^, ^68^ ^]^ Besides, the underoil adhesion performance of the PMPC‐0.1% gel tape is also greater than those of commercially available tapes on the market (Figure S17, Supporting Information).

### 2.3 Investigation of Underoil Adhesion Mechanisms

**Figure 4:**
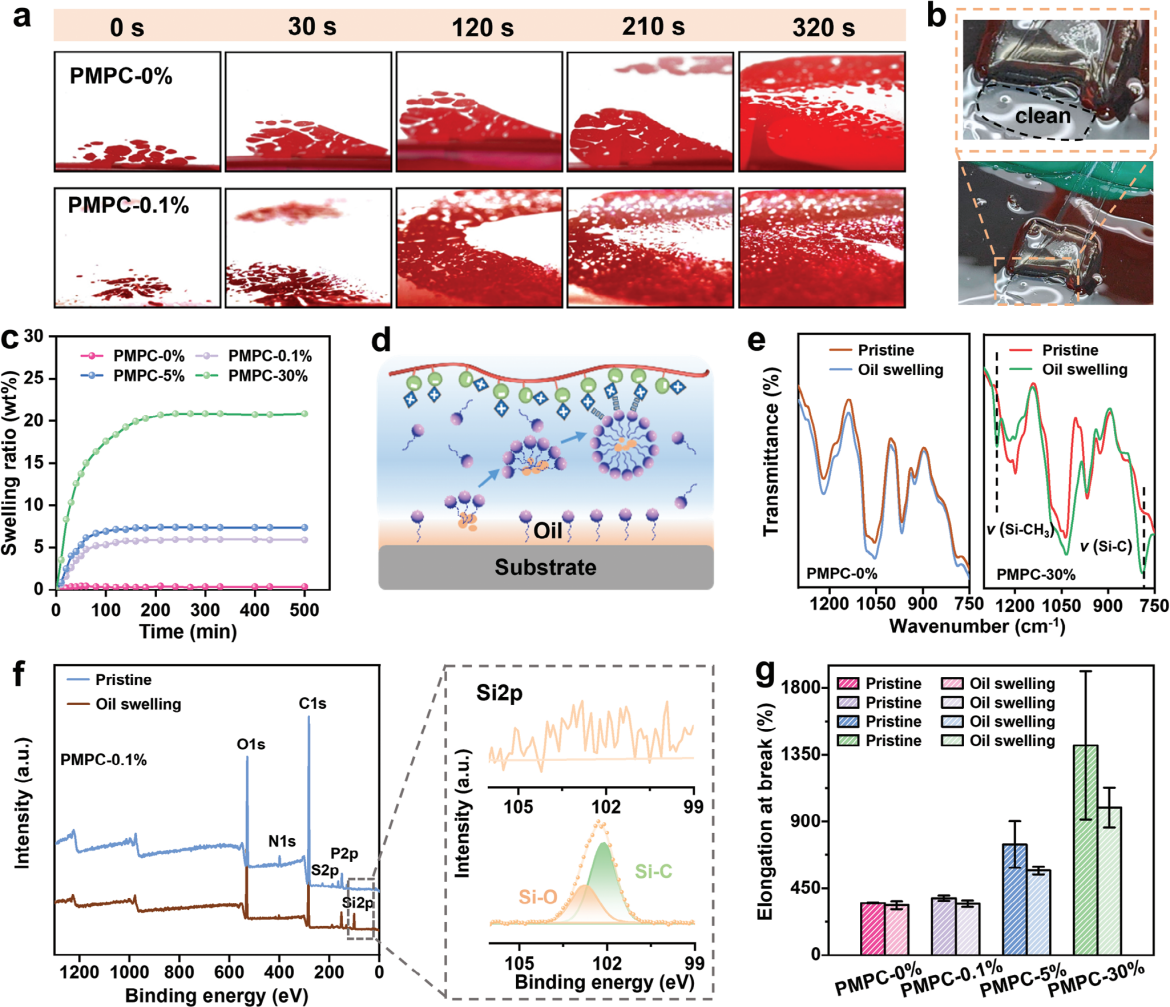
Investigation of underoil adhesion mechanism. a) Photographs to show the contact process of PMPC‐0% gel and PMPC‐0.1% gel to a surface covered with silicone oil. b) Images to show the process of peeling an adhered PMPC‐0.1% gel tape from the oily surface. c) Swelling kinetics of the PMPC‐x% gels in silicone oil. d) Schematic representation of biomimetic oil removal mechanism. e) FTIR spectra of PMPC‐0% gel and PMPC‐30% gel before and after oil swelling. f) XPS spectrum of PMPC‐0.1% gel and fine spectrum of Si before and after oil swelling. g) Elongation of different gels before and after oil swelling. The results are represented as the mean ± SD (n = 3).

To investigate the underlying underoil adhesion mechanism, the contact process of gel and oil‐covered surface is observed by a home‐made setup based on the critical refraction according to previous reports.^[^ ^27^ ^]^ A control group of PMPC‐0% gel without any surfactant is set for comparison. As shown in **Figure** **4**a, the red region in the image is the area that has already covered by red‐dyed gel, whereas the white region is the place still entrapped with the oil. It is found that the gel tape will gradually displace the oil layer and realize direct contact with the surface. Compared to the pure PMPC gel, the PMPC‐0.1% gel contacts with the target surface much quicker; the red region has almost filled the full visual field at 210 s in PMPC‐0.1% gel group, while the control group only cover <50% of the visual field at same time, demonstrating the higher oil removal efficiency of PMPC‐0.1% gel. Besides, a PMPC‐0.1% gel is attached to the steel surface underoil and deposited in silicone oil for 20 min; as presented in Figure 4b, it can be clearly seen that no residual oil exists at the area that gel has just peeled off, confirming the oil at interface has been fully removed.

The oil removal mechanism is investigated. As presented in Figure 4c and Figure S18 (Supporting Information), the PMPC‐0% gel without surfactant shows negligible swelling in silicone oil after equilibrium, demonstrating the outstanding oil resistance of pristine PMPC network. However, the gels containing SB3‐16 show positively increasing swelling behavior in silicone oil with the added surfactant content, indicating that the oil removal process is mainly driven by the SB3‐16.

It has been known that the surfactant preferentially arranges at the interface and the excessive surfactant in bulk phase will form micelles as its concentration exceeds CMC; meanwhile, dynamic exchange exists between free surfactant and the surfactant already assembled in micelle.^[^ ^69^ ^]^ Therefore, in our case, as the gel tape touch an oil‐covered surface, the SB3‐16 surfactants at gel‐oil interface will arrange around the oil droplet to form oil‐filled micelles. After that, the oil‐loaded micelles are transferred into gel via electrostatic interactions between PMPC and SB3‐16 micelles (Figure 4d). As a result, the oil at the interface is removed by micelle transportation, similar to endocytosis.

The uptake of oil in gel is further confirmed by diverse chemical characterizations. As shown in FTIR results, the typical character peaks of silicone oil corresponding to the Si‐CH_3_ (*v =* 1258.72 cm^−1^) and Si‐C (*v =* 789 cm^−1^)^[^ ^70^ ^]^ appear in the spectra of an oil swelling PMPC‐30% gel; meanwhile, these peaks are absent in the spectra of pristine PMPC gel after immersing in oil (Figure 4e). These results confirm the surfactant‐driven oil absorption demonstrated above. As shown in Figure 4f and Figure S19 (Supporting Information), such chemical variation also exists in the results of X‐ray photoelectron spectroscopy (XPS); the characteristic peaks corresponded to the silicone oil (Si─C and Si─O)^[^ ^71^ ^]^ appear in the spectrum of an oil swelled PMPC‐0.1% gel, confirming the uptake of oil again. EDS elemental analysis also proves the presence of silicone oil inside the gel after underoil adhesion (Figure S20, Supporting Information). Also, the mechanical performance of the gel with different surfactant contents before and after oil swelling is compared (Figure S21, Supporting Information). As shown in Figure 4g, the tensile properties of PMPC‐0% gel and PMPC‐0.1% gel show almost negligible change before and after oil swelling, due to the limited oil adsorption; further increasing surfactant content leads to a significant decrease of ultimate strain due to the swelling‐induced brittleness. Therefore, introducing too much surfactant will sacrifice the bulk strength of gel and compromise the underoil adhesion performance. As the result of a balance of oil removal ability and oil‐resistance, the PMPC‐0.1% gel exhibits the best underoil adhesion performance (Figure 3a).

### 2.4 Sustainable Usage of the PMPC‐0.1% Gel Tape

In many practical scenes, remove and reuse of tape is highly required. Sustainable and recyclable usage of adhesive can greatly extend its service life, benefiting the global energy conservation and environmental protection. The adhesive properties of most tape will typically diminish after repeated use due to the oxidation, hydration or contamination of the essential components. This problem is particularly challenging in underoil situation where the adhesives are susceptible to oil contamination.

**Figure 5:**
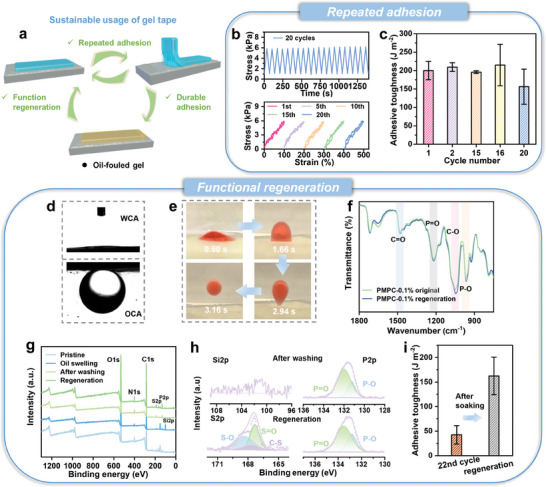
Sustainable usage of the PMPC‐0.1% gel tape. a) Schematic illustration of sustainable usage of the gel tape. b) Cyclic loading curve of PMPC‐0.1% gel at 100% strain and corresponding relationship of maximum stress achieved during each cycle with testing time. c) Adhesive toughness of PMPC‐0.1% gel tape during 20 repeated bonding‐peeling cycles. d) Water contact angle in air and underwater oil contact angle of the PMPC network. e) Photographs to show the excellent underwater‐oil repellency performance of PMPC‐0.1% gel. f) FTIR spectra of original and regenerated PMPC‐0.1% gel. g) The XPS spectra of PMPC‐0.1% gel at different stages (original, after use, after water washing, and after regeneration); h) Corresponding XPS fine spectra of the related Si, S, and P elements. i) The adhesive toughness of the gel tape after 22 cycles and the regenerated gel. The results are represented as the mean ± SD (n = 3).

Here, we successfully realize a combination of i) durable adhesion in each single use, ii) repeated use, and iii) function regeneration after the exhausted use (**Figure** **5**a). As shown in Figure 5b, the tensile performance of the PMPC‐0.1% gel remains highly stable with negligible hysteresis during successive loading‐unloading process. Thanks to the low hysteresis and oil‐resistance characteristics of the bulk gel, the tape can be repeated used for up to 20 bonding‐peeling cycles without obviously declined adhesive toughness (Figure 5c). The adhesive toughness of gel begins declining obviously after 20 cycles, due to the gradually consumption of micelles and the inevitable oil‐contamination of gel surface.

Nevertheless, such exhausted PMPC‐0.1% gel tape after extreme use can be easily cleaned just by water rinsing (Figure S22, Supporting Information). As shown in Figure 5d, the PMPC backbone exhibits i) ultra‐high hydrophilicity in air (water contact angle in air: 3.6^°^ ± 0.26^°^) and ii) underwater superoleophobicity (oil contact angle in water: 147^°^ ± 8.63^°^), due to superior water binding capability of zwtterionic groups.^[^ ^72^, ^73^, ^74^, ^75^, ^76^, ^77^ ^]^ Benefiting from these, the flat silicone oil layer attached on the PMPC‐0.1% gel surface would shrink into a droplet and detach from the surface within ≈3 s after contacting water, demonstrating the excellent underwater oil repellency (Figure 5e). Such oil dewetting behavior of PMPC‐0.1% gel in water also occurs in other oil systems (Figure S23, Supporting Information). To better regenerate the adhesion function, the cleaned gel tape is further immersed into SB3‐16 solution to replenish fresh surfactants. The FTIR curve of the regenerated gel tape is overlapped with that of the original one, confirming the successful regeneration in chemical composition (Figure 5f). Similarly, as revealed by the XPS results, the water can effectively remove the silicone oils from gels as the signals related to Si element disappear; after absorbing fresh surfactants, the XPS signals of the regenerated gel recover to be comparable to those of the original one (Figure 5g,h). As shown in Figure 5i, the adhesive toughness of the regenerated gel almost recovers to the original level, demonstrating the successful regeneration.

## 3 Conclusion

In summary, we designed a biomimetic gel tape that can achieve robust adhesion to various substrates in diverse oil environments. The highly viscous and stubborn oil droplets adhered on the target surface can be efficiently removed through the cooperation of zwitterionic surfactant SB3‐16 and PMPC network, mimicking the endocytosis process. The maximum adhesive toughness of the obtained gel achieves 2446.86 J m^−2^; such value outperforms the underoil adhesion performance of commercially available tapes and even higher than many reported underwater adhesion cases. Furthermore, the adhesive can realize a combination of i) durable adhesion in each single use, ii) repeated use, and iii) function regeneration after the exhausted use, demonstrating the excellent sustainability. We believe the biomimetic zwitterionic gel tape developed here offers new method in liquid removal and promotes the development of high efficient adhesives for using in wet environments.

## 4 Experimental Section

### Materials

2‐methacryloyloxyethyl phosphorylcholine (MPC, 98%) was purchased from Bide Pharmatech Co., Ltd. N,N‐methylene‐bis‐acrylamide (MBAA), 2959 (photoinitiator, 98%), 3‐(N, N‐Dimethylpalmitylammonio) propanesulfonate (SB3‐16, 98%), mineral oil, and n‐Decane (98%) were purchased from Aladdin Reagent Co., Ltd. Glycerol (AR, ≥99.0%) was supplied by Sinopharm Chemical Reagent Co., Ltd. Silicone oil and n‐Hexane were supplied by Hangzhou Jigong Biotechnology Co., Ltd. Colza oil was purchased from local supermarkets. Oil red O was purchased from Hefei BASF Biotechnology Co., Ltd. All of the chemicals were used as purchased without further purification. Deionized water was obtained from a Millipore water purification system.

### Preparation of the Gel Tape

Monomer (MPC), chemical cross‐linker (MBAA) photo‐initiator (2959), and surfactant (SB3‐16) were added in a mixture solvent of glycerol and water; the solution was treated with ultrasonication for a period of time to obtain completely dissolved precursor solution. The precursor solution was injected into a glass mold and exposed to 365 nm UV light for 3.5 h to get gel.

### Characterizations

^1^H nuclear magnetic resonance (NMR) spectra of samples were conducted on ANANCE III 400 MHz. TEM images of the nanocapsules were obtained with a Bruker HT7700 EXALENS at 120 kV at room temperature. Fourier transform infrared spectroscopy (FTIR, Nicolet 6700) was utilized to characterize the chemical composition of the gel. Transmittance of adhesives by UV–vis Spectroscopy. Detection of adhesive adsorption to interfacial oil layers using x‐ray photoelectron spectroscopy (US, Thermo Scientific ESCALAB Xi+). The surface morphologies and elemental distributions of the hydrogels were characterized using scanning electron microscopy (Hitachi S4700) and EDS. Formation of micelles in precursor fluids with different surface‐activity contents observed by DLS (UK, Malvern Zetasizer Pro). The CA‐200 machine was utilized to measure the contact angle of the gels at room temperature.

### Mechanical Tests

The tensile test of the samples was carried out on a universal testing machine (Instron 5966). Rheological frequency scans were performed on disc‐shaped samples of 8 mm diameter by using a DHR rheometer (TA Instruments). To measure the adhesive toughness, the gels were cut into rectangular shapes and corresponding length and width were recorded; 90° peeling test was performed, with a peeling speed of 50 mm min^−1^. The adhesive toughness is calculated through the formula of Г = force/width (J m^−2^). For adhesion of oil‐contaminated surface, the substrate was into silicone oil before pasting the gel tape by applying a pressure of 250 Pa for different period. It should be noted that, in order to prevent the bulk gel from deforming during the peeling process and thus influence the test results, a stiff PET film is attached to one side of the gel. All substrates were wiped clean with ethanol and water before use.

### Molecular Simulation

The density functional theory (DFT) calculations were performed using the program DMol3 in Material Studio (Accelrys, USA) to investigate the optimized geometries for the interactions of A with B and the binding energies (*E*_binding_) between A and B. The general gradient approximate (GGA) system was carried out, and the BLYP functions and DNP basis set were used. The *E*_binding_ could be calculated using the following equation:

(2) $$E_{\mathrm{binding}}=E_{A+B}-E_{A}-E_{B}$$

where *E*_binding_ (kJ mol^−1^) is the binding energy of A with B; *E*_A+B_ is the total energy of the A/B system; *E*_A_ is the energy of the A; *E*_B_ is the energy of the B molecule.

### Interface Observation

The contact of the gel with the substate under‐oil was observed by a home‐made setup using critical refraction reported.^[^ ^27^ ^]^ A trapezoidal prism (≈51 g) gradually approached the gel (10 × 10 × 1 mm) in silicon oil from the top until the normal force reached ≈2 N, during this process, the contact image of the gel to the prism surface is observed from an angle between the critical refraction angles of oil and the gel.

### Swelling Ratio

The swelling ratio *S* of the adhesive in oil is calculated by:

(3) $$S=\left(W_{\mathrm{oil}}-W_{i}\right)/W_{i}$$

where *w*_oil_ is the weight of the gel after equilibrium in oil, and *w*_i_ is the original weight of the gel before swelling.

### The Regeneration of Gel

The used tape was rinsed with DI water to remove the absorbed oil based on the underwater oil repellency mechanism; then the gel was incubated in the surfactant solution to load fresh surfactant. The surfactant/glycerol/water fraction of the incubated solution is consistent with that of the precursor for synthesizing gel.

### Statistical Analysis

Statistical analysis of data presented in this work had a sample size n = 3–5 and was presented as the mean ± SD.

## Conflict of Interest

The authors declare no conflict of interest.

## Supporting information



Supporting Information

## Data Availability

The data that support the findings of this study are available from the corresponding author upon reasonable request.
