# Supplementary material for: Endocytosis‐Inspired Zwitterionic Gel Tape for High‐Efficient and Sustainable Underoil Adhesion
Source: Adv Sci (Weinh). 2024 Sep 9;11(42):2407501. doi: 10.1002/advs.202407501 (PMC11558084; doi:10.1002/advs.202407501)
Supplement: Supplementary file 1 — Supporting Information [file ADVS-11-2407501-s001.docx]

**Supporting Information**

***for***

Endocytosis-inspired zwitterionic gel for high-efficient and sustainable underoil adhesion

*Yueman Tang^1^, Mengjie Si^1^, Yan-jie Wang^1^, Jiahui Zhou, Yuming Deng, Kaishun Xia, Dong Zhang, Si Yu Zheng*, Jintao Yang*

Corresponding author: Prof. Si Yu Zheng, [zhengsiyu@zjut.edu.cn](mailto:zhengsiyu@zjut.edu.cn)

**Supporting materials**

| PMPC-x% | MPC [g] | SB3-16 [g] | MBAA [g] | 2959 [g] | Water [g] | Glycerol  [g] |
| --- | --- | --- | --- | --- | --- | --- |
| PMPC-0% | 1.173 | 0 | 0.001 | 0.01 | 0.6 | 0.5 |
| PMPC-0.1% | 1.173 | 0.001173 | 0.001 | 0.01 | 0.6 | 0.5 |
| PMPC-0.5% | 1.173 | 0.005865 | 0.001 | 0.01 | 0.6 | 0.5 |
| PMPC-1% | 1.173 | 0.01173 | 0.001 | 0.01 | 0.6 | 0.5 |
| PMPC-5% | 1.173 | 0.05865 | 0.001 | 0.01 | 0.6 | 0.5 |
| PMPV-30% | 1.173 | 0.3519 | 0.001 | 0.01 | 0.6 | 0.5 |

Table S1. Detailed formulation of PMPC-x% adhesives.

To prepare the precursor solution, 1.173 g of zwitterionic monomer MPC, 0.001 g of MBAA and 0.01 g of 2959 were added in a mixture solvent of glycerol and water (in a 4:6 volume ratio), and surfactants SB3-16 of different mass fractions (relative to the mass of the MPC) were added after complete dissolution, sonication for a period of time to obtain a completely dissolved solution. The precursor solution was injected into a prepared glass mold and exposed to 365 nm UV light for 3.5 h for polymerization. The prepared zwitterionic gel adhesive was named PMPC-x%.

**Supporting figures**

**
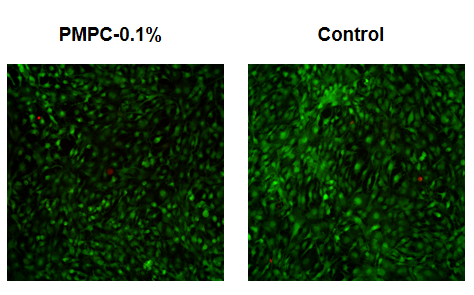
**

Figure S1. The biocompability of the PMPC-0.1% gel and the control group.


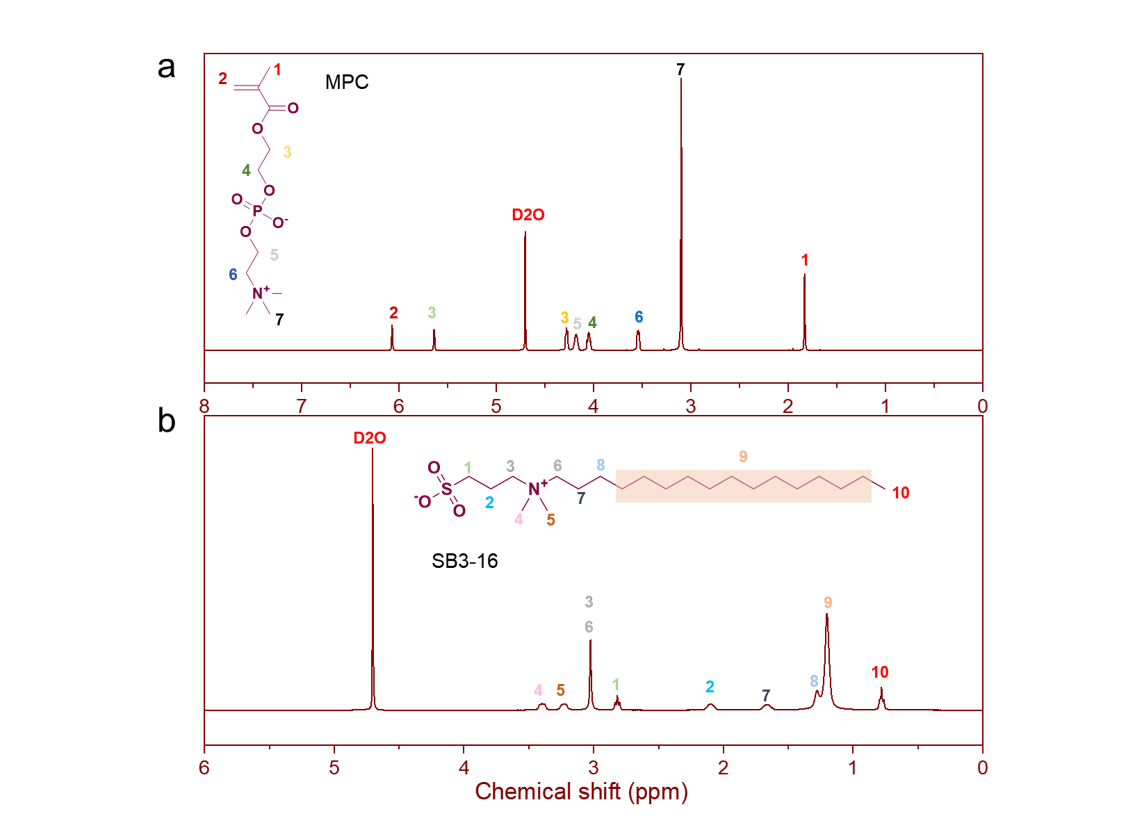


Figure S2. ^1^H NMR spectra of a) MPC monomer and b) SB3-16.


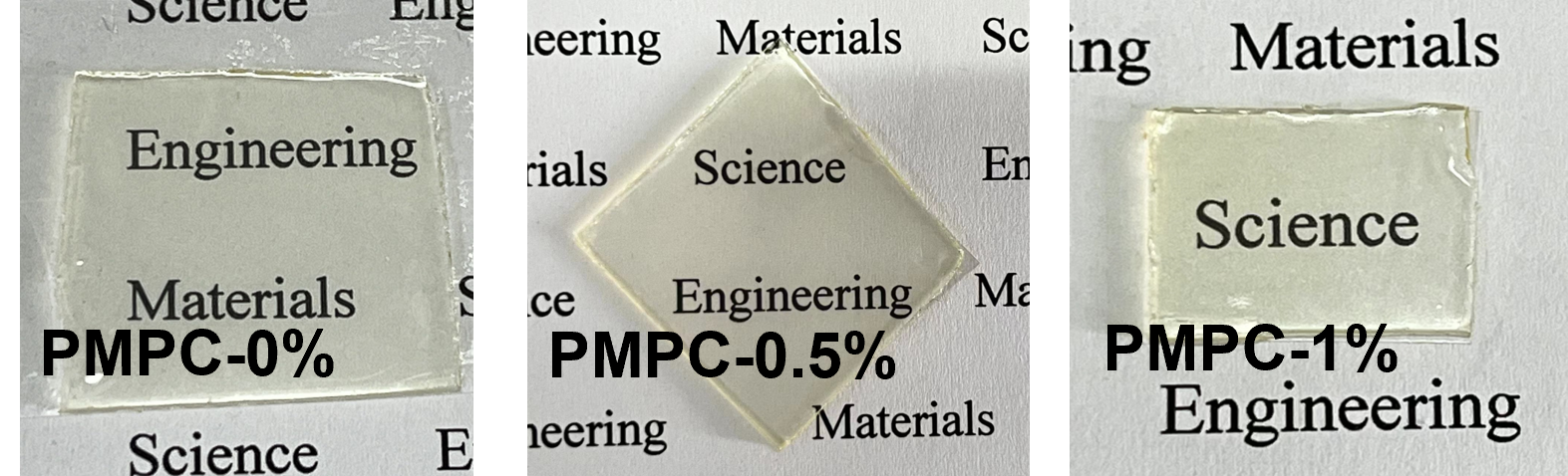


Figure S3. Photographs of PMPC-0%, PMPC-0.5% and PMPC-1% to show the appearance of the gels with different surfactant content.


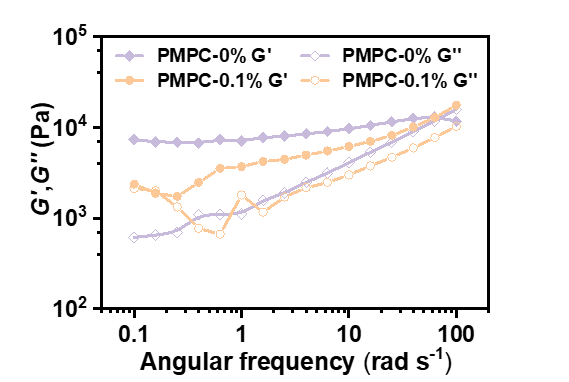


Figure S4. Rheological behavior of the PMPC-0% gel and PMPC-0.1% gel.

Figure S5. pH value of the PMPC-x% precursor solution.


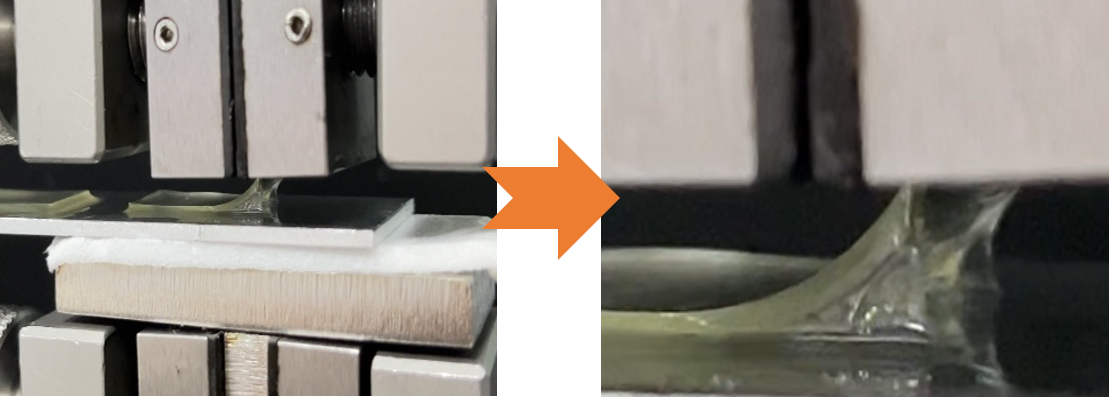


Figure S6. Photographs to show the peeling process of zwitterionic gel tape on a tensile tester.


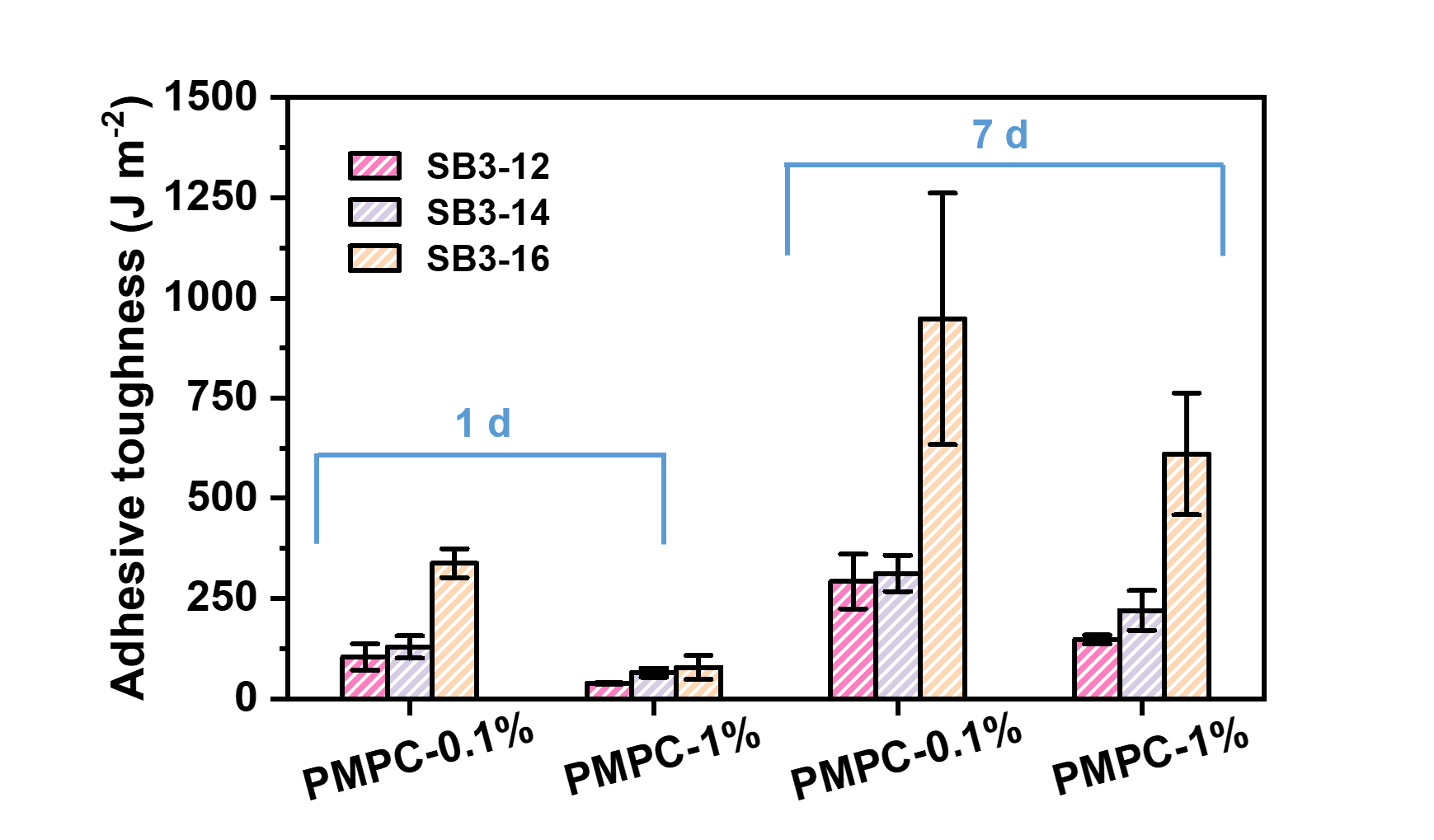


Figure S7. The adhesion toughness of the gel tape containing surfactant with different alkyl chain length to stainless steel surface in silicone oil.


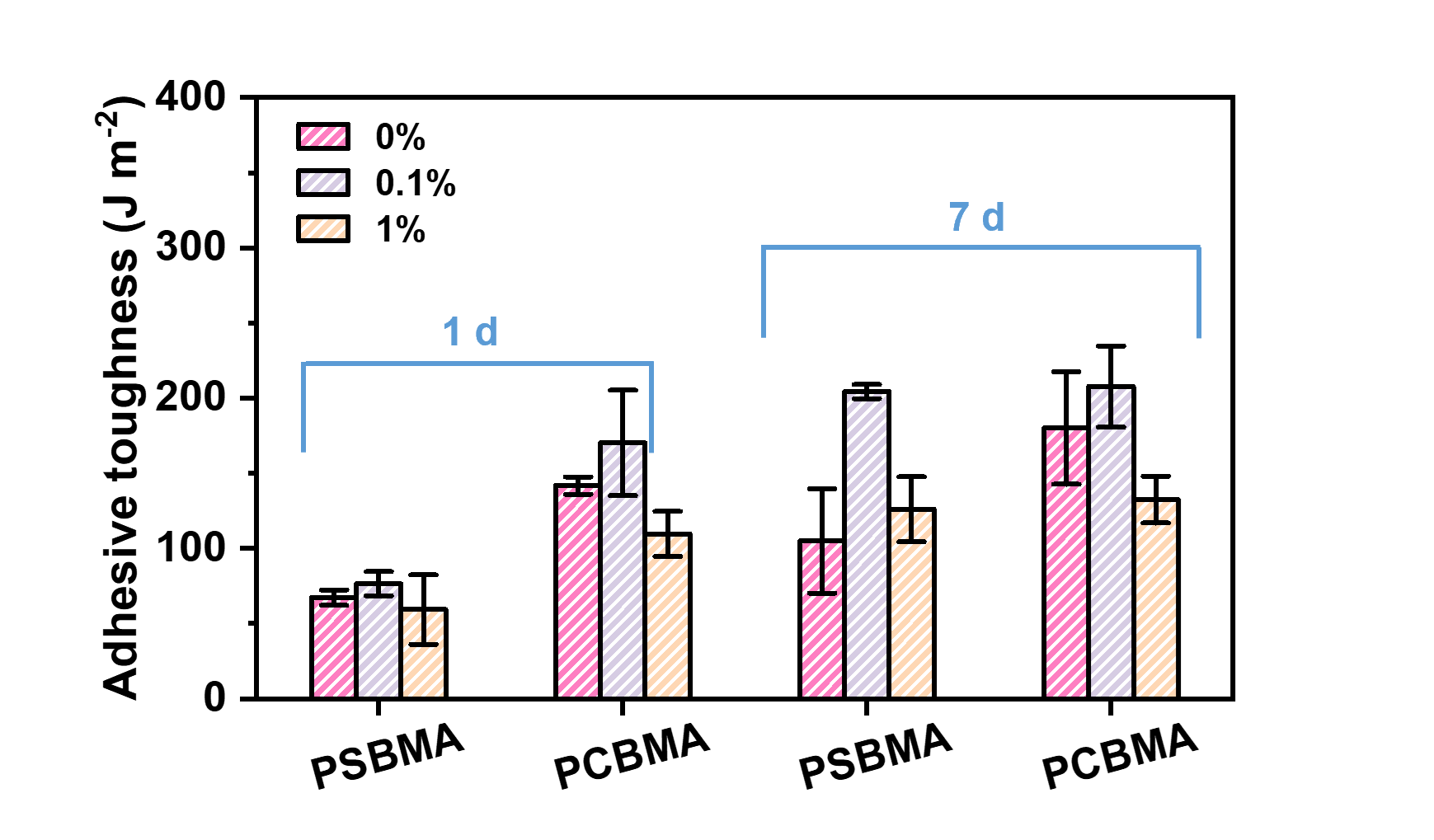


Figure S8. Underoil adhesion performance of the gel tapes based on different polyzwitterions to stainless steel in silicone oil.


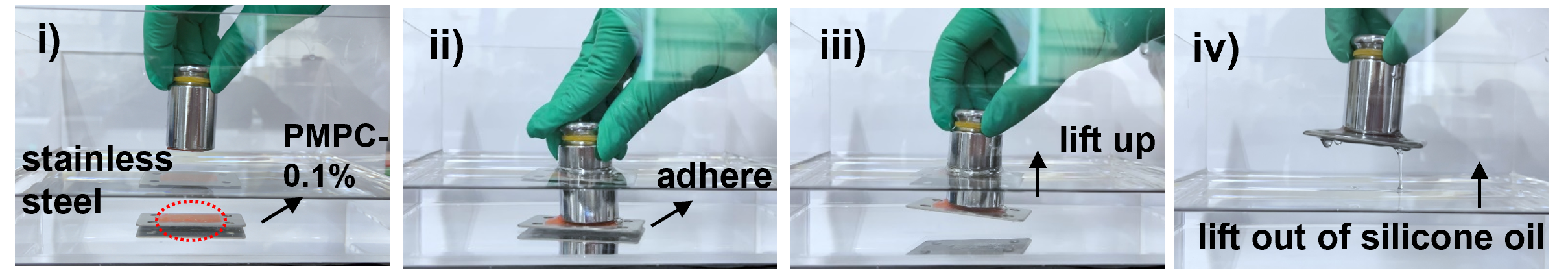


Figure S9. Picking up a stainless steel substrate attached with PMPC-0.1% adhesive on its surface by a weight from the bottom of the oil-filled tank.


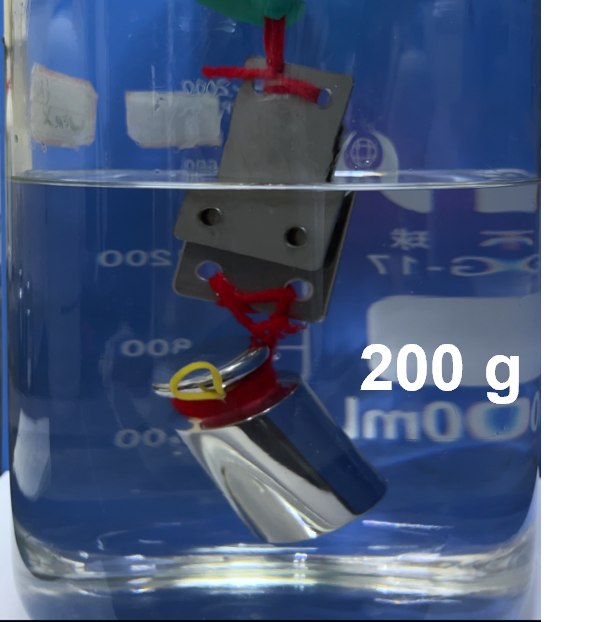


Figure S10. Two stainless steel sheets are bonded with a piece of PMPC-0.1% gel underoil, which can bear 200 g weight in oil.


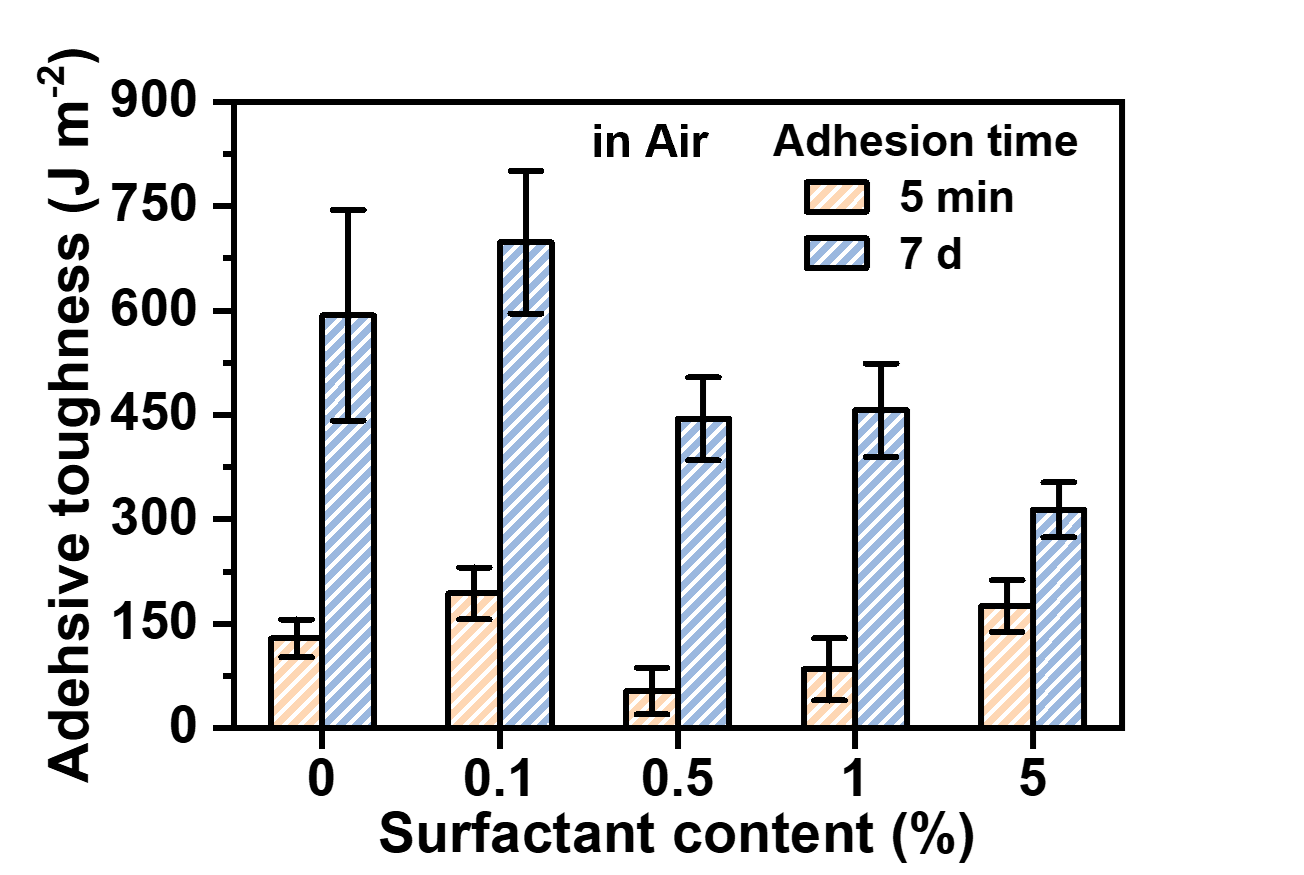


Figure S11. Short and long-time adhesion of PMPC-0.1% gel to stainless steel in air.


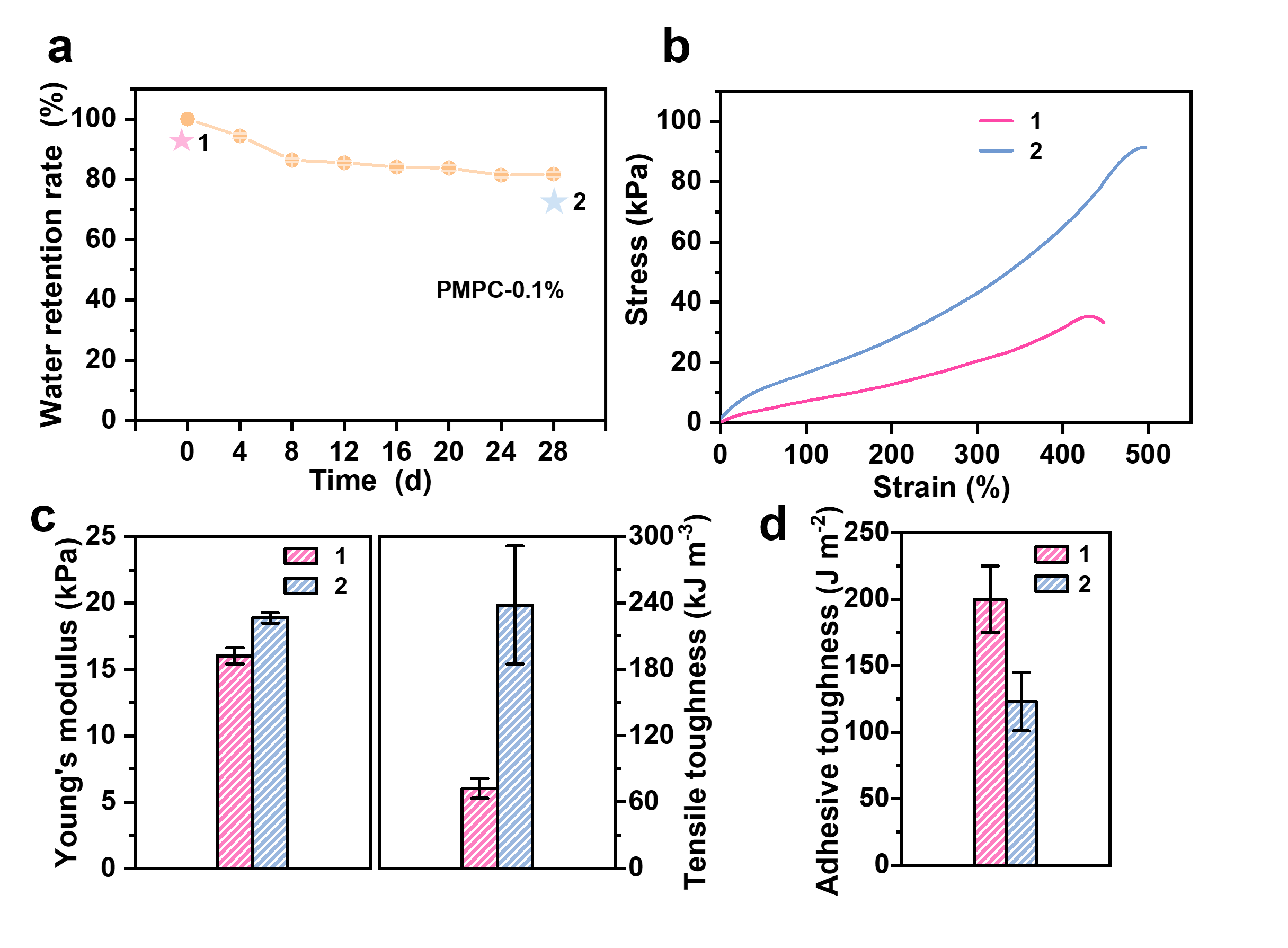


Figure S12. a) Water retention rate of PMPC-0.1% gel tested by depositing the gel in air for different intervals. b) Stress-strain curves of original PMPC-0.1% gel and the gel after depositing in air for 28 days; c) corresponding Young's modulus and tensile toughness. d) Adhesive toughness of original PMPC-0.1% gel and the gel after depositing in air for 28 days; adhesion time: 1 day.


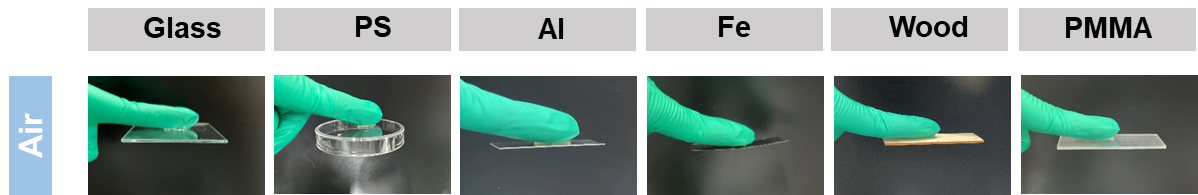


Figure S13. Photographs to show the robust adhesion of PMPC-0.1% gel to diverse surfaces in air.


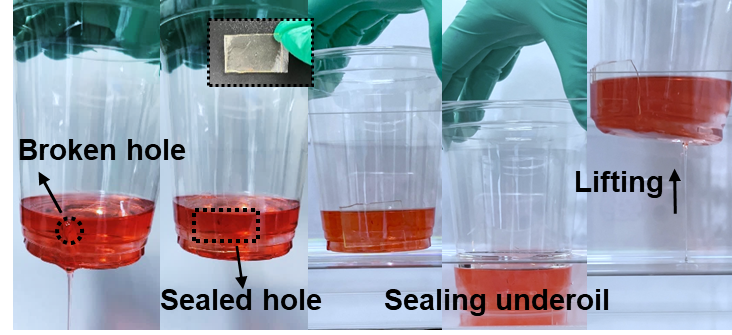


Figure S14. Photos of sealed plastic bottles immersed in oil-filled tanks.


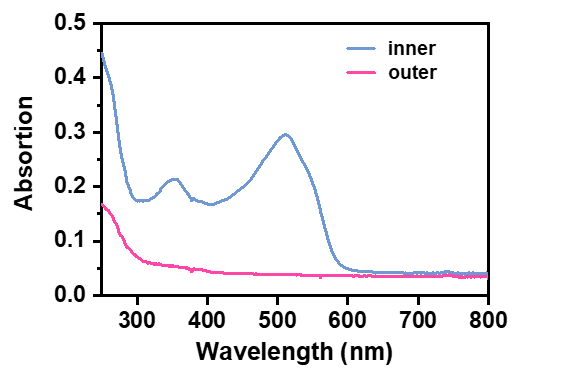


Figure S15. UV absorption curves of oil inside and outside the broken bottle after underoil sealing.


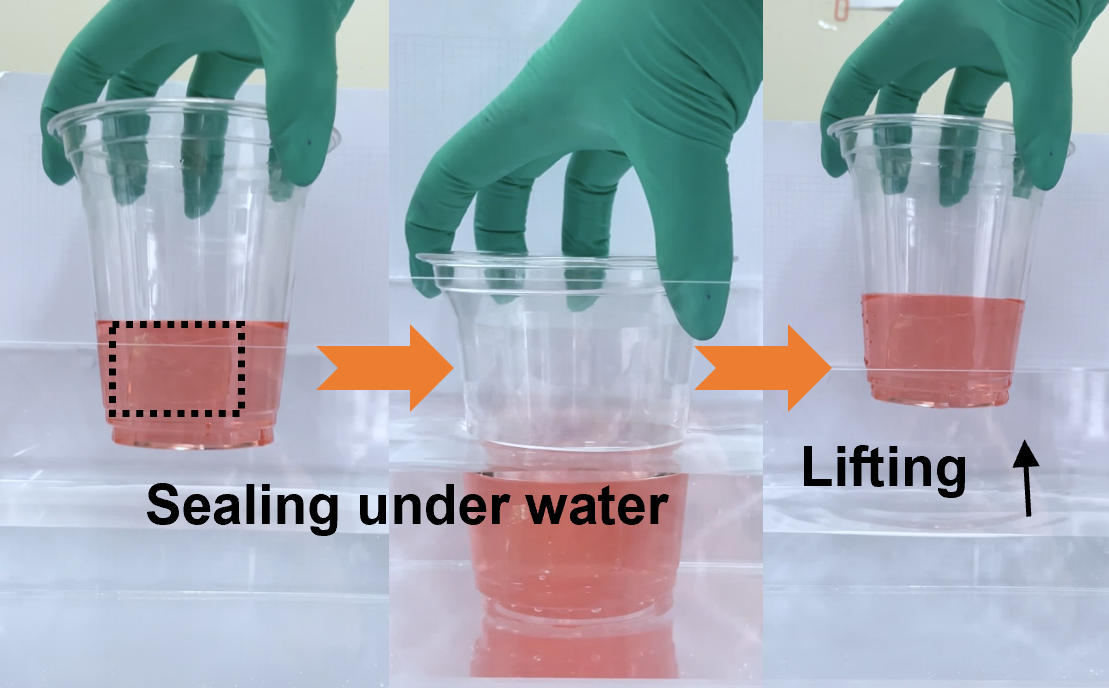


**Figure S16**. PMPC-0.1% adhesive seals plastic bottles in water.


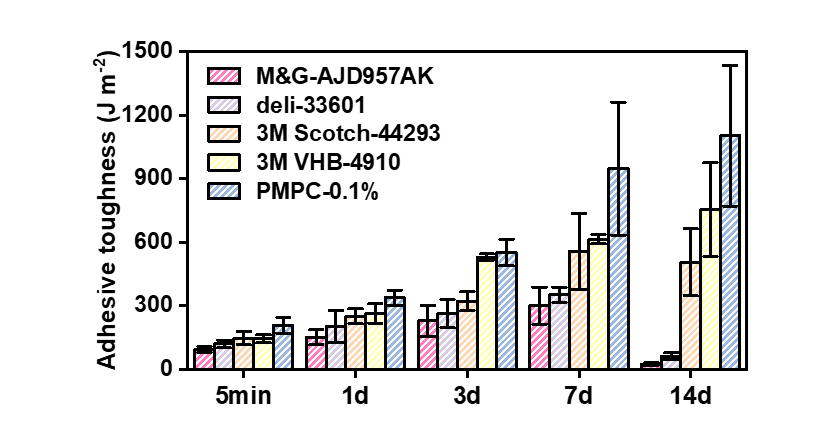


Figure S17. Adhesive toughness of PMPC-0.1% gel and various commercial tapes under silicone oil over different time.


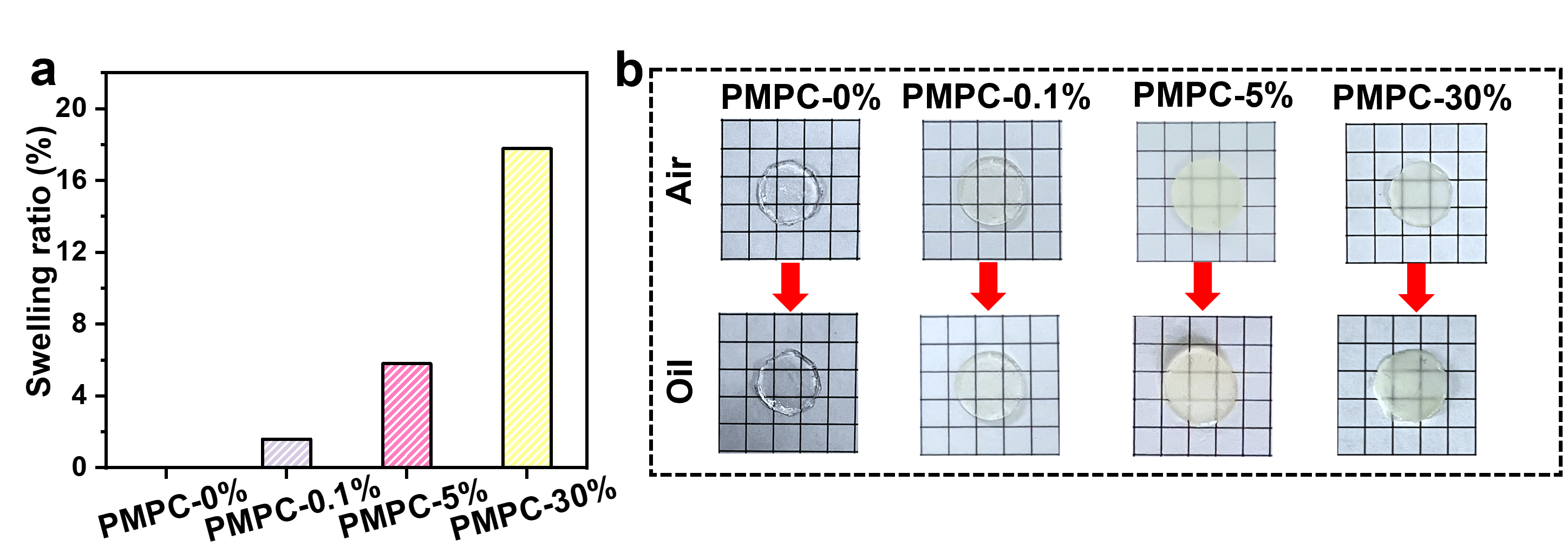


Figure S18.a) Swelling ratio of gels with different surfactant contents in silicone oil. b) The photos show appearance of the gels before and after oil swelling.


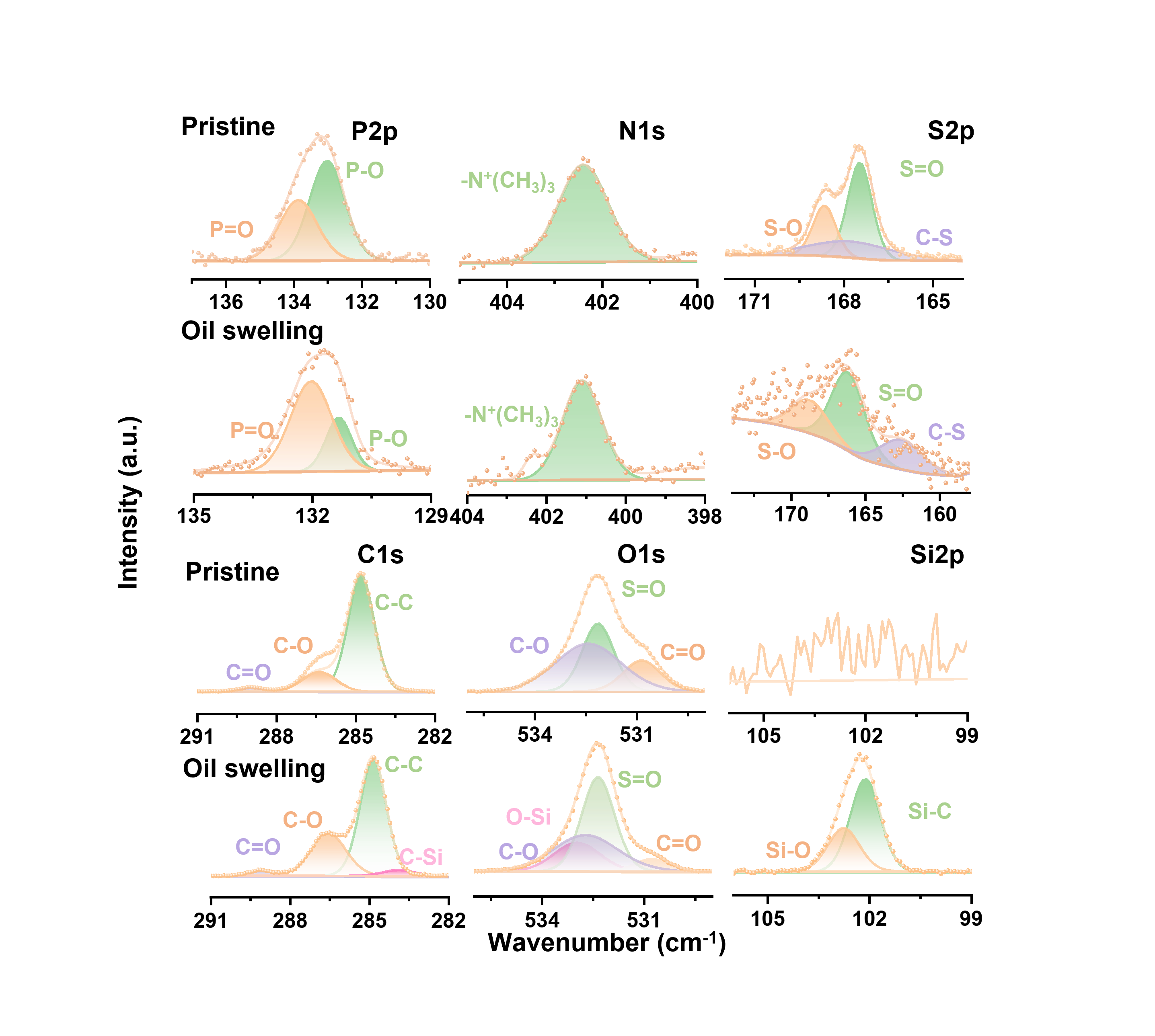


Figure S19. XPS Fine spectra of PMPC-0.1% gel before and after oil swelling.


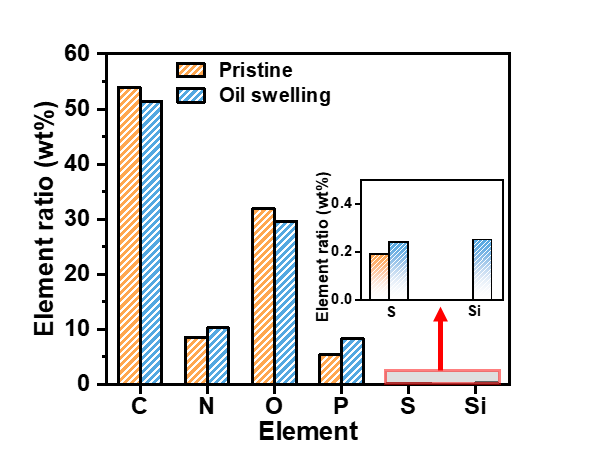


Figure S20. EDS elemental analysis of PMPC-0.1% gel tape before and after oil swelling.


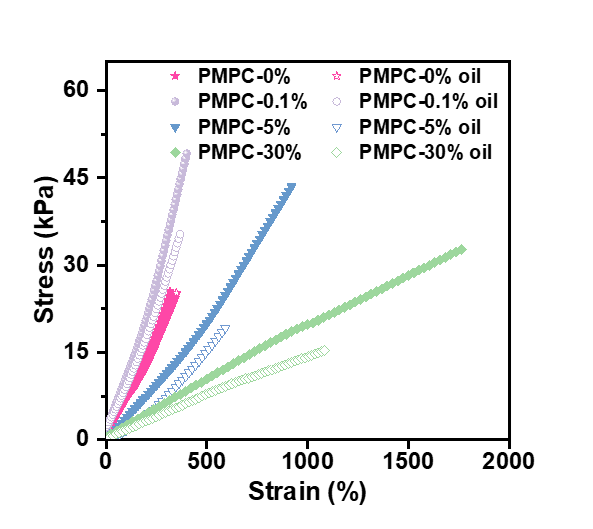


Figure S21. Stress-strain curves of different gels before and after oil swelling.


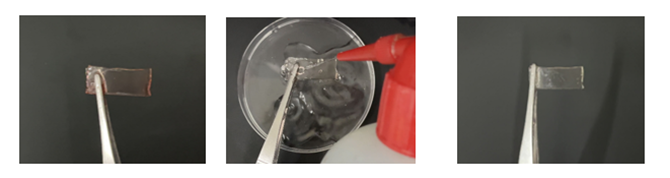


Figure S22. Rinsing of oil (dyed with oil red O) on the surface of PMPC-0.1% adhesive.


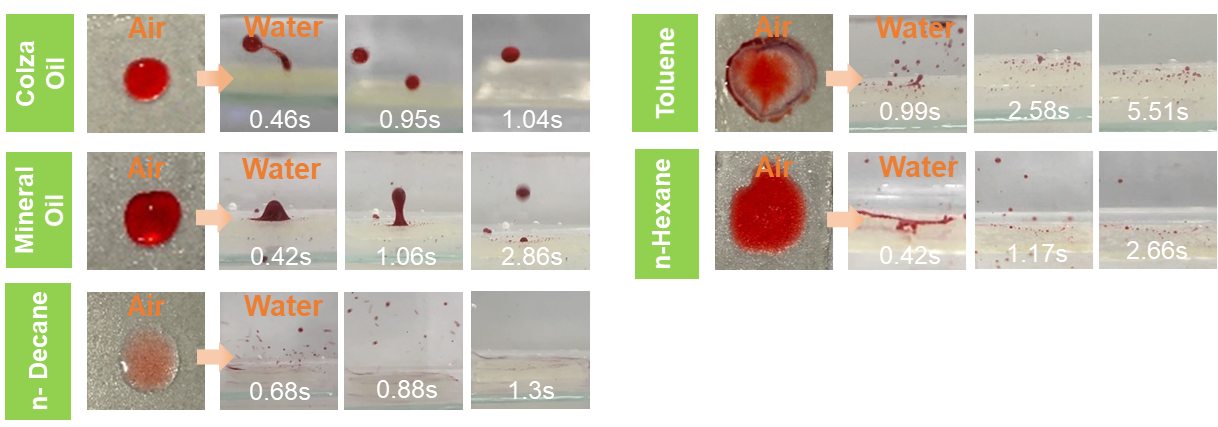


Figure S23. Photographs of underwater detachment of different kinds of oils on the surface of PMPC-0.1% gel.
